# Vagus nerve stimulation using a miniaturized wirelessly powered stimulator in pigs

**DOI:** 10.1038/s41598-022-11850-0

**Published:** 2022-05-17

**Authors:** Iman Habibagahi, Mahmoud Omidbeigi, Joseph Hadaya, Hongming Lyu, Jaeeun Jang, Jeffrey L. Ardell, Ausaf A. Bari, Aydin Babakhani

**Affiliations:** 1 Electrical and Computer Engineering Department, University of California Los Angeles, Los Angeles, CA USA; 2 Department of Neurosurgery, University of California at Los Angeles, Los Angeles, CA USA; 3 UCLA Cardiac Arrhythmia Center, University of California Los Angeles, Los Angeles, CA USA; 4 UCLA Neurocardiology Research Program of Excellence, University of California Los Angeles, Los Angeles, CA USA; 5 Molecular, Cellular and Integrative Physiology Program, University of California Los Angeles, Los Angeles, CA USA

**Keywords:** Electrical and electronic engineering, Cardiac device therapy

## Abstract

Neuromodulation of peripheral nerves has been clinically used for a wide range of indications. Wireless and batteryless stimulators offer important capabilities such as no need for reoperation, and extended life compared to their wired counterparts. However, there are challenging trade-offs between the device size and its operating range, which can limit their use. This study aimed to examine the functionality of newly designed wirelessly powered and controlled implants in vagus nerve stimulation for pigs. The implant used near field inductive coupling at 13.56 MHz industrial, scientific, and medical band to harvest power from an external coil. The circular implant had a diameter of 13 mm and weighed 483 mg with cuff electrodes. The efficiency of the inductive link and robustness to distance and misalignment were optimized. As a result, the specific absorption rate was orders of magnitude lower than the safety limit, and the stimulation can be performed using only 0.1 W of external power. For the first time, wireless and batteryless VNS with more than 5 cm operation range was demonstrated in pigs. A total of 84 vagus nerve stimulations (10 s each) have been performed in three adult pigs. In a quantitative comparison of the effectiveness of VNS devices, the efficiency of systems on reducing heart rate was similar in both conventional (75%) and wireless (78.5%) systems. The pulse width and frequency of the stimulation were swept on both systems, and the response for physiological markers was drawn. The results were easily reproducible, and methods used in this study can serve as a basis for future wirelessly powered implants.

## Introduction

During the past decade, implantable medical devices (IMDs) have proven useful in clinical treatments of hypertension^1^, pain^2^, neurological disorders^3^, and inflammation^4^ through electrophysiological stimulation. Some examples of successful demonstrations of IMDs include flexible near-field wireless optoelectronics such as subdermal implants^5^, wireless, batteryless, and fully implantable electrical neurostimulation in freely moving rodents^6,7^, and wireless spinal stimulation system for ventral activation of the rat cervical spinal cord^8^. Technical developments have led to the increasing use of neuromodulation in the management of various disorders using less invasive peripheral nerve modulation methods such as vagus nerve stimulation (VNS) and dorsal root ganglion stimulation^9–11^.

There are limited studies that have examined wireless stimulation of peripheral nerves, including the vagus nerve. VNS therapy has been FDA approved for use in reducing the frequency of seizures in epileptic individuals and for the treatment of depression^12–14^. The vagus nerve contains afferent (80%) and efferent (20%) fibers^15^. The cell bodies of afferent vagal nerve bers are housed in the inferior vagal ganglion, projecting centrally to the central nervous system, where their processes terminate primarily in the nucleus tractus solitarius (NTS)^15,16^. From the NTS, there are direct afferent projections to the locus coeruleus and raphe nuclei which widely project to structures including the thalamus, cerebellum, hypothalamus, amygdala, insula, cingulate, and frontal cortical areas. Activating this pathway may explain the cognitive and behavioral changes induced by VNS^17^. The descending efferent fibers from the nucleus ambiguous and nucleus dorsalis in the brainstem bridge visceral organs, including the lungs, heart, and gastrointestinal tract, with the central nervous system^18^. Increased efferent vagus nerve activity leads to a slowing heart rate via inhibition of the sinoatrial node by the release of acetylcholine, the main vagal nerve neurotransmitter^18^. The effect of VNS on the descending pathway allows us to monitor the device’s functionality and to quantitatively compare its performance with conventional systems.

Current battery-powered systems face complicated problems due to the bulky nature of the devices, such as the need for frequent charging in clinical studies^6,19–23^.

Patient populations highly prefer implants with small sizes which do not need recharging or replacement^24^. The wireless power transfer (WPT) technology enables experiments in a more naturalistic environment than their tethered counterparts^6,19^. The most commonly used WPT technique is near field high frequency (3–30 MHz) coupling^25–28^. The ultrahigh frequency (300–3000 MHz) mid-field coupling suffers from higher tissue attenuation and polarization alignment^29,30^. However, a circularly polarized field increases the WPT efficiency and decreases the misalignment sensitivity^3^. Other approaches, such as capacitive^31^ and ultrasonic^24,32^ WPT, are being explored, but they still need to show reliable operation in large animals^33^.

In this work, we have tested a wireless VNS stimulator working at 13.56 MHz ISM band and cuff electrodes in pigs to show feasibility. Using a 12 turn double-layer circular coil on a flexible polyimide substrate, the need for an oversized power harvesting coil is alleviated. The customized power harvesting chip only consumes 6.2 $\upmu$W of power and enables demodulation of the data and power harvesting. Furthermore, an accurate method was proposed to maximize the efficiency of transmission and reception of power. Using cuff electrodes, the wirelessly powered implant (WPI) was connected to the vagus nerve . Using electrochemical impedance spectroscopy (EIS), the tissue impedance before stimulation was measured and verified. The performance of the device was shown using a small amount of peak power (0.1–1 W), and operational distances from 50 to 100 mm were achieved. Our findings indicated specific absorption rates that were orders of magnitude lower than the safety regulation limit. This paper was the first demonstration of wirelessly powered and controlled VNS in pigs with more than 5 cm operating range. A comparison with hard-wired conventional stimulators was drawn for the same animals. The methodology for WPT, wireless control, and verification of performance can also be used as a broad platform for other wireless IMDs. Using these methods and devices, we demonstrated VNS efficacy in anesthetized pigs.

## Results

### System design

**Figure 1:**
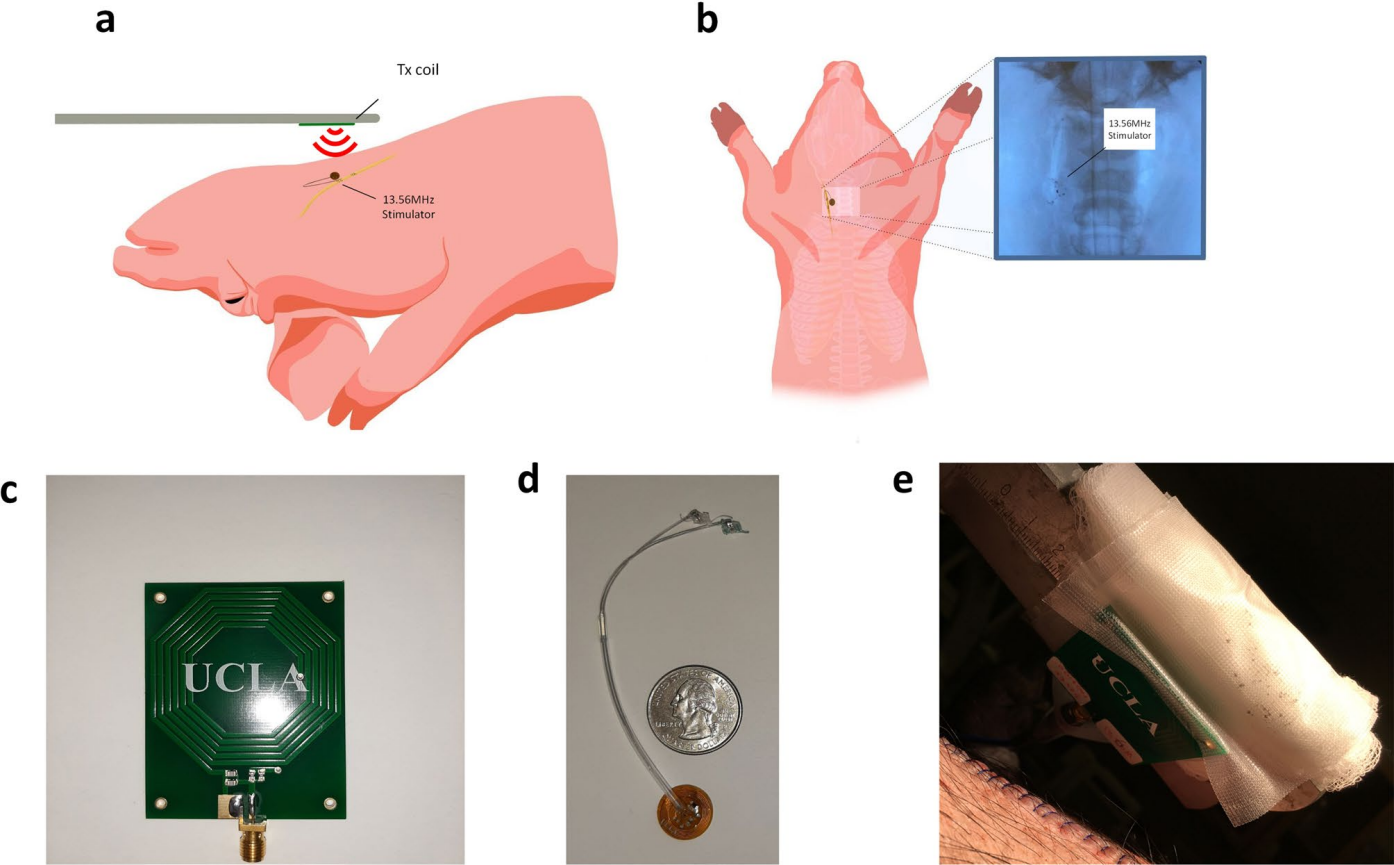
Conceptual representation of the VNS system and photos of the designed stimulator. (**a**) Conceptual illustration of wirelessly powered VNS setup. (**b**) X-ray image of the device after implantation. (**c**) 45 mm diameter Tx coil used for transmitting power and data. (**d**) Picture of stimulator compared to US quarter. (**e**) Picture of the pig after suturing the incision with the flexible stimulator inside the neck.

Conceptual representation of the VNS system is shown in Fig. 1a. The miniaturized flexible stimulator can be implanted subcutaneously and wirelessly powered and controlled at the 13.56 MHz ISM band. The stimulator weighs 483 mg and 81 mg with and without cuff electrodes, respectively, and the receiver coil is 13 mm in diameter. Figure 1b shows an X-ray image of the stimulator inside the animal. The Tx coil is shown in Fig. 1c and Supplementary Fig. S1, which show the front and backside, respectively. The Tx coil has a diameter of 45 mm and is connected to an RF signal generator (E4428C, Hewlett Packard Inc.) to transmit power and data. The signal generator, along with an optional power amplifier (ZHL-20 W-13 +, Mini-Circuits Inc.) is used for wireless powering of the stimulator, and the internal pulse modulation feature of the RF signal generator can be used to set the frequency and pulse width of stimulation. The sample setup and operation of the Tx coil are presented in Supplementary video S1. The flexibility and small form factor of the implantable device allow subcutaneous operation and natural movement of the neck after implantation. The dimensions and placement of the device after incision closure are demonstrated in Fig. 1d, e, respectively.

### Stimulator design

**Figure 2:**
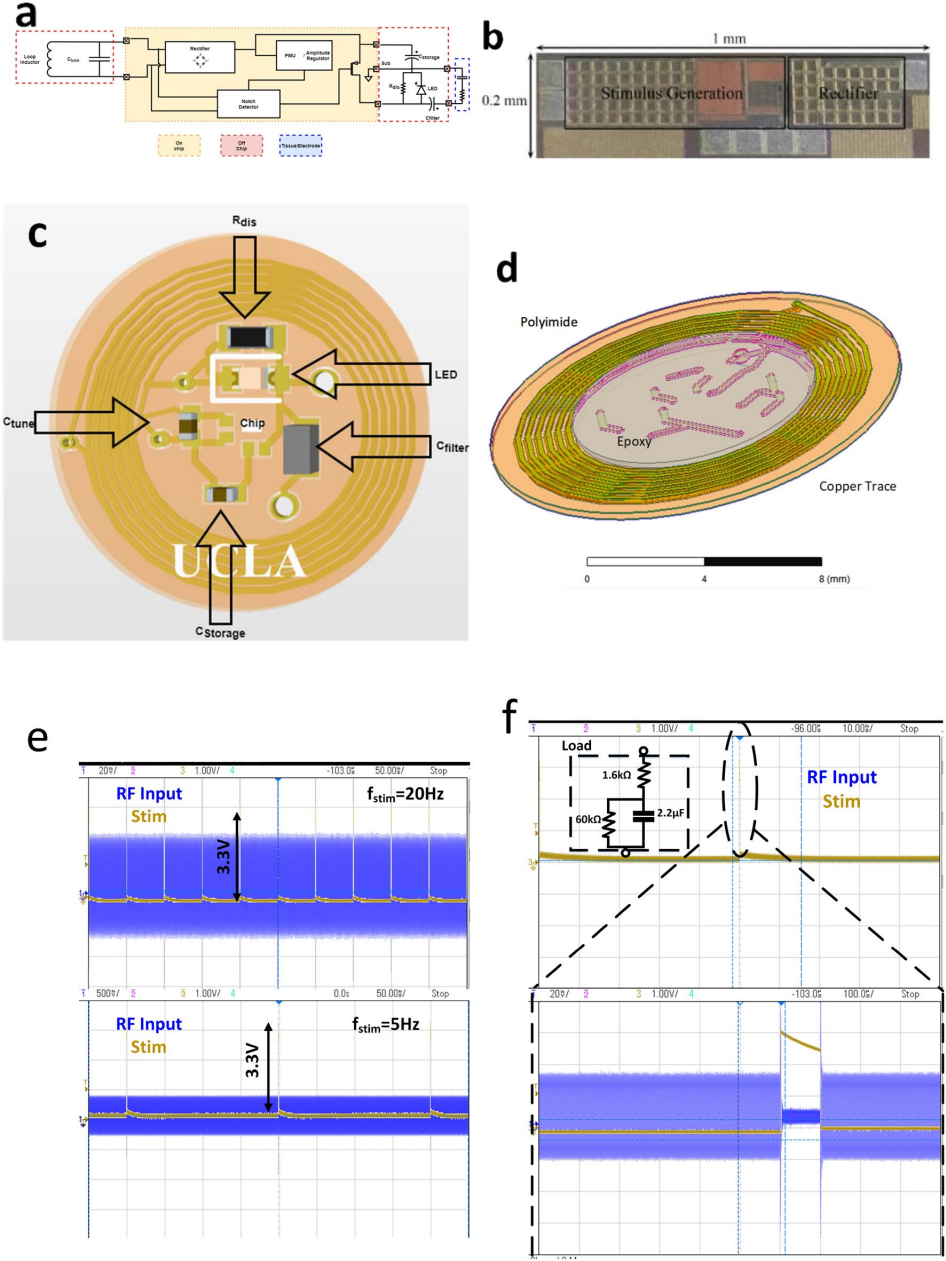
Stimulator design. (**a**) Block diagram of the designed chip. (**b**) Picture of fabricated IC. (**c**) Assembled components on the top side of stimulator PCB. (**d**) 3D model of Rx coil. (**e**) The Measured response of the stimulator to 5 Hz and 20 Hz stimulation. (**f**) The measured response of stimulator to 100 $\upmu$s stimulation across the modeled load.

Most of the stimulation devices operate based on current-controlled stimulation (CCS), voltage-controlled stimulation (VCS), or switched-capacitor stimulation (SCS)^27,34–36^. CCS offers high safety; however, it consumes a vast majority of power to comply with worst-case impedances^34–36^. VCS offers higher efficiency at the cost of lower safety since there is no control over the charge injected into the tissue. In this paper, an SCS scheme is presented. The SCS has a simple design similar to VCS and high safety and controllability similar to CCS, making it favorable for low-power designs^27,28,34,36^. SCS systems have efficiency levels between VCS and CCS^28,34^. The operator controls the stimulation by varying the pulse width and frequency. A schematic diagram of the chip is presented in Fig. 2a, and detailed circuitry is described in Ref.^28^. The chip is fabricated in 180 nm standard complimentary-metal-oxide-semiconductor (CMOS) technology. The chip has a small area of 0.2 mm $\times$ 1 mm, including the pad area shown in Fig. 2b. Five discrete components are used on the stimulator printed circuit board (PCB). The rectifier resonance frequency can be tuned using a tuning capacitor ($C_{tune}=47$ PF). The power is continuously harvested on a discrete storage capacitor ($C_{charge}=22~\upmu$F). To block the DC voltage, a series filtering capacitor ($C_{filter}=10~\upmu$F) and parallel discharge resistor ($R_{dis}=47~{\text {K}}\Omega$) are assembled at the output. An optional green light-emitting diode (LED) is also included at the output to visually identify the stimulation. Figure 2c illustrates the PCB with an Rx coil and SMD components on the top side. Figure 2d presents a 3D image of the Rx coil fabricated on a 25 $\upmu$m flexible polyimide substrate with 1 Oz copper traces. The Rx coil has six turns on each side, which enabling power harvesting at wavelengths much larger than its dimensions ($>1000\times$). Biocompatible epoxy (EPO-TEK, MED301) is placed on the PCB for encapsulation and insulation from blood. Figure 2e represents a sample output voltage in response to 100 $\upmu$s stimulation at frequency of 20 Hz and 5 Hz. The PCB needs about 20 ms to balance the charge. By choosing a smaller filter capacitance, this time can be reduced however the penalty is less charge being delivered to the tissue. Figure 2f shows the 100 $\upmu$s pulses, and it should be considered that the stimulator was loaded with the equivalent circuitry measured from the EIS of the tissue. The chip activates the output whenever there is a notch in the incoming RF waves, as shown in Fig. 2f, and wirelessly transmitted notches are controlled by an external RF signal generator (E4428C, Hewlett Packard Inc.). The voltage of the stimulator is regulated between the 3.7 and 2.6 V by on-chip voltage limiter and control loop. The stimulator, including the protective epoxy, weighs only 483 mg and 81 mg with and without cuff electrodes. The light weight of the stimulator is mainly due to the elimination of the battery and incorporation of small SMD components. The cuff electrodes (PerenniaFLEX Model 304) and SMD components are assembled on the PCB using silver epoxy (EPO-TEK, H20E). All samples were put in phosphate-buffered saline (PBS) three days before the implantation to test for the leakage and isolation of the device.

### Wireless power transfer link design

**Figure 3:**
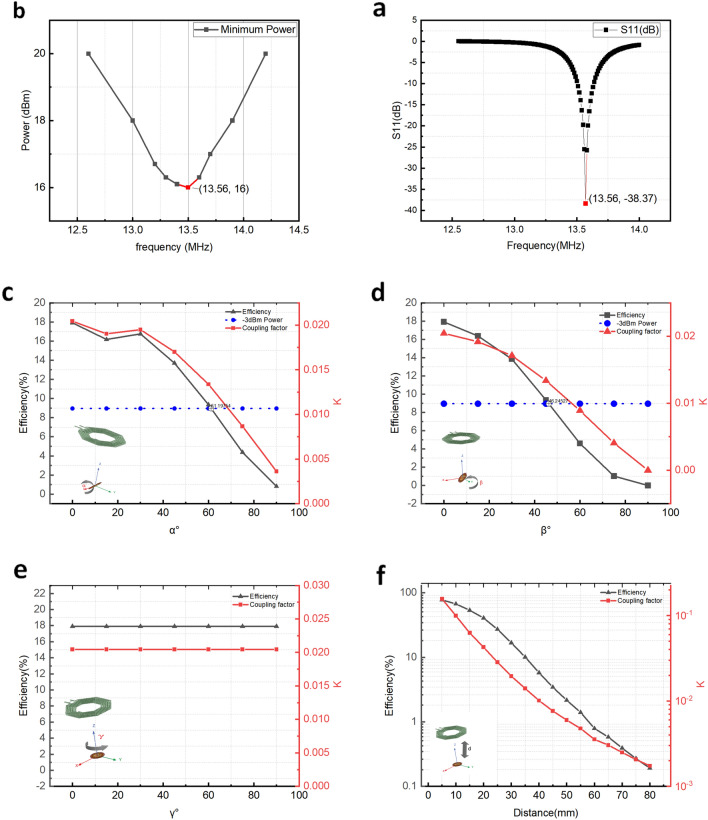
Inductive power transfer design. (**a**) The image shows the minimum required external power to operate the stimulator at a distance of 50 mm. (**b**) Measured $S_{11}$ (matching) for the Tx coil, which presents better than $-$38 dB matching. Simulated efficiency and coupling factor (K) fluctuations in response to variations in the (**c**) x-axis ($\alpha$), (**d**) y-axis ($\beta$), (**e**) z-axis ($\gamma$), and (**f**) distance (**d**).

The 13.56 MHz Tx coil is fabricated using a 1.6 mm FR4 substrate with six turns on each side. The Tx coil has a diameter of 45 mm and measured quality factor (Q$_t$) is 39. On the receiver side, for maximum current delivery, the inductor is resonated with a high-quality factor (Q$>200$) 47 pF capacitor. Unlike the transmitter coil, the inductance cannot be directly measured due to the high parasitic inductance of probes and the relatively small size of the receiver coil. Before matching the Tx coil, using a novel method, in a separate setup, the minimum power for LED to start blinking using an unmatched (wide-band) Tx coil was measured at different carrier frequencies. The unmatched Tx coil transmits pulses of 100 $\upmu$s every one second. At a fixed frequency, the power is increased slowly until the LED starts blinking. This indicates that at least 2.7 V is accumulated on the storage capacitor, and power of 80 $\upmu$W is delivered by the chip. The frequency at which minimum power is required to turn on the LED is the resonance frequency of the coil. The result is shown in Fig. 3a and verifies that a carrier frequency of 13.56 MHz requires the minimum power. To ensure maximum power delivery from the signal generator, the transmitter coil was matched to 50 $\Omega$ at the same resonance frequency. The S11 measured using the VNS (PNA-L network analyzer) Model N5230C shows better than $-$38.4 dB matching, and therefore the terminal efficiency is higher than 99.99$\%$. The measured $S_{11}$ versus frequency is shown in Fig. 3b.

The simulated quality factor for Rx coil ($Q_r$) is 65.2. The link efficiency is a function of mutual coupling (K), according to Eq. (1)^37^. To maximize link efficiency, mutual coupling needs to be maximized^38^. Using HFSS (Ansys Inc.) simulations, variations in coupling with respect to distance and angular misalignment were simulated, and the point at which coupling decreases by $\frac{1}{\sqrt{2}}$ ($-$3 dBm power) was found. Figure 3c, d show that the link (at $d=30$ mm) has $-$3 dBm tolerance up to 62$^{\circ }$ and 46$^{\circ }$ for $\alpha$ and $\beta$ misalignment, respectively. The coil has negligible sensitivity for $\gamma$ misalignment, as shown in Fig. 3e. The robustness toward $\gamma$ misalignment is expected since the direction of the magnetic field toward the transmitter coil is not changed. In the next simulation, the effect of the distance between the Rx and Tx coils on the coupling and efficiency was investigated, and the results are shown in Fig. 3f. It can be observed that coupling is proportional to the inverse square of the distance. The robustness to angular misalignment is due to the larger size of the transmitter coil and usage of a circular shape for the receiver, which guarantees the maximum quality factor.

1 $$\begin{aligned} \eta \approx \frac{K^2Q_{t}Q_{r}}{(1+\sqrt{1+K^2Q_{t}Q_{r}})^2} \end{aligned}$$

### EIS

**Figure 4:**
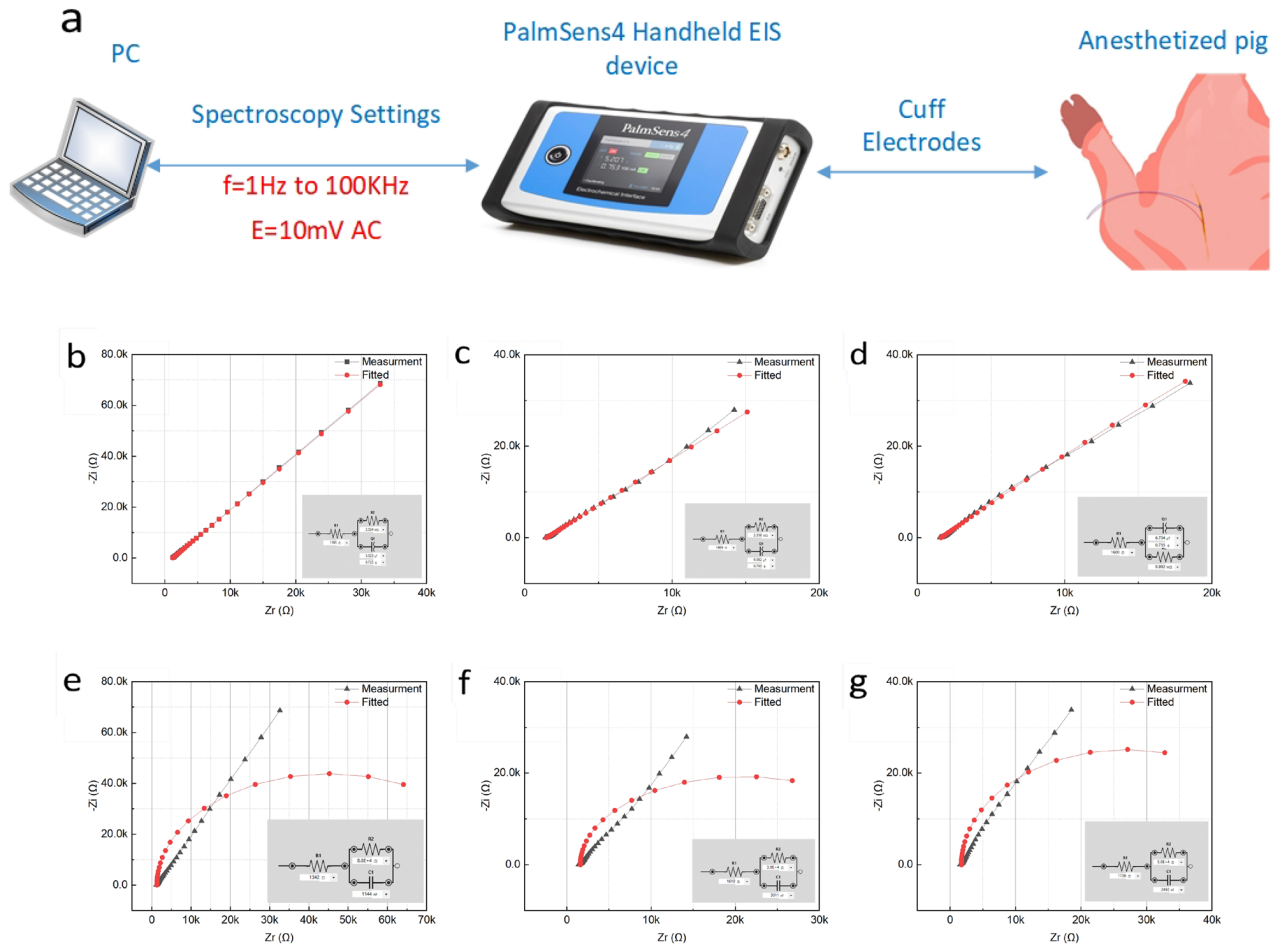
Presentation of EIS for connection verification and load calculation. (**a**) EIS measurement setup along for the 1 Hz to 100 kHz range using a 10 mV AC signal. (**b–d**) Nyquist plots of the impedance of tissue and fitted circuit using CPE and two resistors for circuit fitting. (**e–g**) Nyquist plots of the impedance of tissue and fitted circuit using a capacitor and two resistors for circuit fitting.

To verify the electrode contacts had proper contact with the tissue and the stimulator load impedance was within its operational range, the impedance of tissue was measured before implantation. Before implanting the stimulators in the open incision for each pig, nerve impedance was measured using a Palmsens4 EIS device, and the impedance phase and magnitude were obtained by PSTrace software. Impedance spectroscopy was completed by using a 10 mV AC voltage from 1 Hz to 100 kHz. Figure 4a shows the setup along with connections. The extracted Nyquist plots for the measured impedance and fitted circuits for each pig are shown in Fig. 4b–d. For the best circuit fitting, a constant phase element (CPE) along with two resistors ($R_{1}$, $R_{2}$) was used. The average modeling errors for Q, n, $R_{1}$, and $R_{2}$ are 2.6$\%$, 0.76$\%$, 0.75$\%$, and 3.21$\%$, respectively. To reconstruct the tissue impedance with off-the-shelf components at low and medium frequencies, the CPE is simply replaced with a capacitor. The newly fitted impedances are represented in Fig. 4e–g. The average values for $R_{1}$, $R_{2}$, and $C_{1}$ are 1.56 K$\Omega$, 58.85 K$\Omega$, and 2.22 $\upmu$F, with average modeling errors of 4.3$\%$, 13.7$\%$, and 7.0$\%$, respectively. The performance of each stimulator was verified by the average impedance load before implantation.

### Vagus nerve stimulation

**Figure 5:**
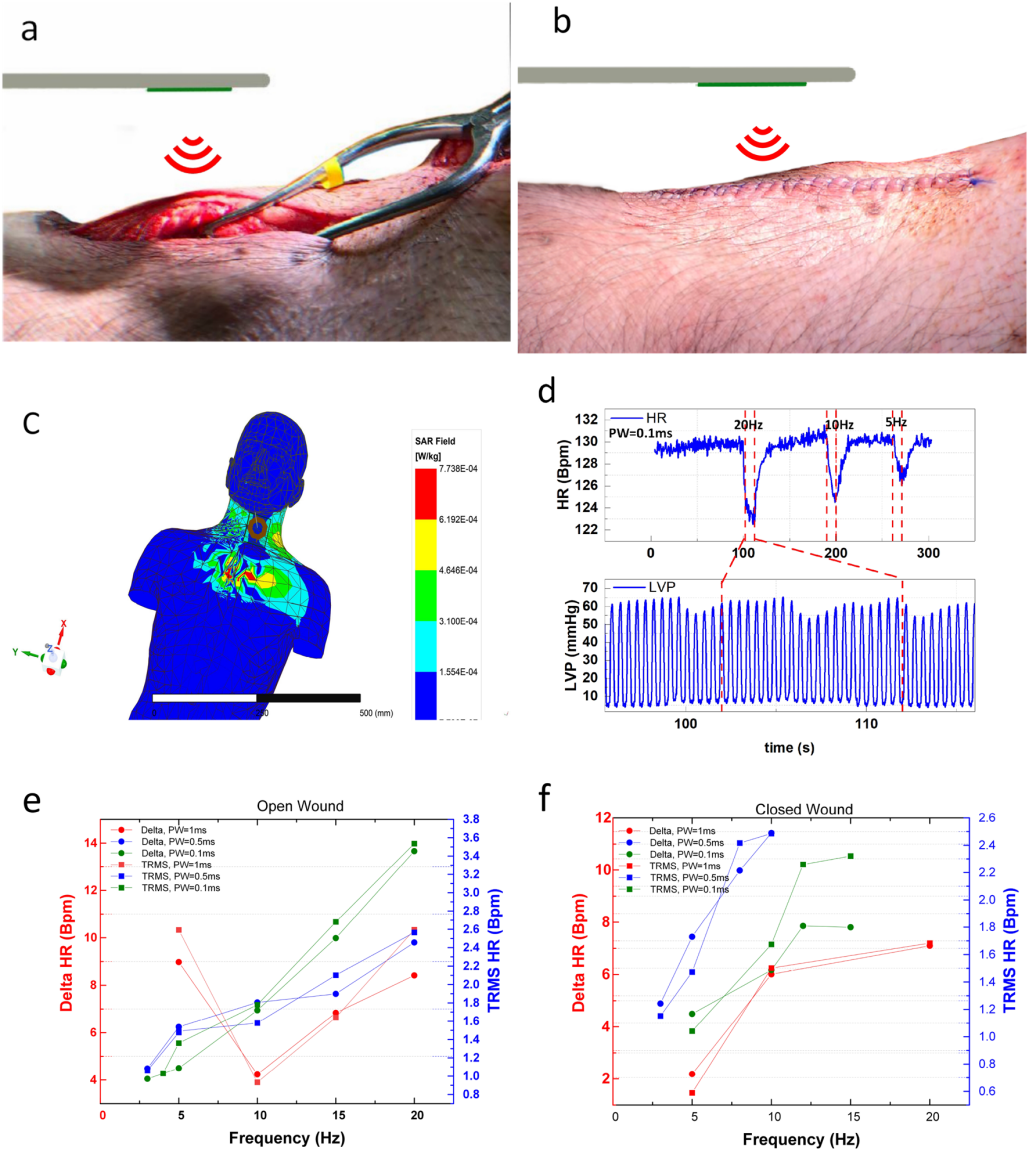
Animal experiments using WPI device. (**a**) The placement of WPI inside the animal for open incision experiments. (**b**) Sutured incision with WPI inside the animal and external powering. (**c**) Simulated SAR at 13.56 MHz with 0.1 W of peak power. (**d**) Change in HR and periodicity of LVP in response to stimulation at various frequencies with a constant pulse width of 0.1 ms. $HR_{Delta}$ and $HR_{TRMS}$ respond to different frequencies and pulse widths using WPI while the incision is (**e**) open and (**f**) sutured.

In vivo studies were carried out acutely in three pigs (*Sus scrofa*, n = 3, female, adult 40–44 kg). The WPI devices were implanted on the right side of the neck using standard surgical techniques, and they were powered up using 0.1 W peak power at 13.56 MHz. The stimulation duration was 10 s. The frequency and pulse width were swept from 3 to 20 Hz and 0.1 to 1 ms accordingly. For the conventional constant current stimulator, the current was set to 3 mA. To compare both systems (wireless and conventional) in the same setting and to evaluate the wireless system’s performance in a fully implanted state, nerve stimulations were performed in both open and sutured incisions. Figure 5a, b show the placement of the device before and after suturing the incision, respectively. The operation distance could be extended from 50 to 100 mm by increasing the power to 1 W. The simulated SAR is obtained from HFSS software (Ansys Inc), and it shows for future human studies SAR with 0.1 W of external power is 0.77 mW/Kg, as shown in Fig. 5c. In this work SAR is four orders of magnitude smaller than the 10 W/Kg limit specified by IEEE Std C95.1-2005. Vitals of animals were monitored using 3-lead electrocardiography, pulse oximetry, arterial blood pressure, end-tidal carbon dioxide, and temperature. Heart rate (HR) was calculated by the periodicity of left ventricle pressure (LVP). During the experiments, we observed a hemodynamic response for the HR while stimulation was in process. Furthermore, it was observed that changing the frequency and pulse width of stimulation could produce changes in HR. Hemodynamic responses are presented in Supplementary videos S2, and S3 for open and sutured incisions, respectively. An example of the HR and LVP response at different frequencies of 5 Hz, 10 Hz, and 20 Hz with a constant pulse width of 0.1 ms is shown in Fig. 5d. As expected from Fig. 5d, a higher frequency of stimulation induced a stronger response in HR. In this study, we calculated the maximum change in HR by defining $HR_{Delta}$ using Eq. (2), and the average changes in HR were also calculated using the true root mean square (TRMS) of HR by Eq. (3). Figure 5e, f show the response of $HR_{Delta}$ and $HR_{TRMS}$ to WPI during one set of sweeps in open and sutured incisions, respectively. Figure 5e, f show that both $HR_{Delta}$ and $HR_{TRMS}$ follow the same trends while the frequency and pulse width are swept. The significance of this response and its correlation to frequency and pulse width are studied in the next section.

2 $$\begin{aligned} HR_{Delta}= & {} HR_{max}-HR_{min} \end{aligned}$$

3 $$\begin{aligned} HR_{TRMS}= & {} \sqrt{\frac{\int _{T_{start}}^{T_{end}}(HR-HR_{Avg})^2 dt}{T_{start}-T_{end}}} \end{aligned}$$

#### Comparison

Before testing the WPI, all the animals were tested by a conventional wired stimulator interfaced to a constant current photoelectric stimulus isolation unit (Grass S88 and PSIU6, Grass Instruments, Warwick, Rhode Island). The conventional Grass stimulator is a bulky (48.3 cm $\times$ 13.4 cm $\times$ 31.8 cm) device that needs to be hard-wired to the electrodes. A total of 84 stimulations by the WPI device (28 stimulations in sutured and 56 stimulations in open incisions) and 36 (all open incisions) nerve stimulations by the conventional system were performed. The conventional VNS stimulation system in 75$\%$ of stimulations caused a significant heart rate reduction in open-incision ($P < 0.05$, paired *t*-test). A total of 71.4$\%$ and 78.5$\%$ of WPI device stimulations led to significant heart rate reduction in the open incision and sutured incision conditions, respectively ($P < 0.05$, paired *t*-test).

**Figure 6:**
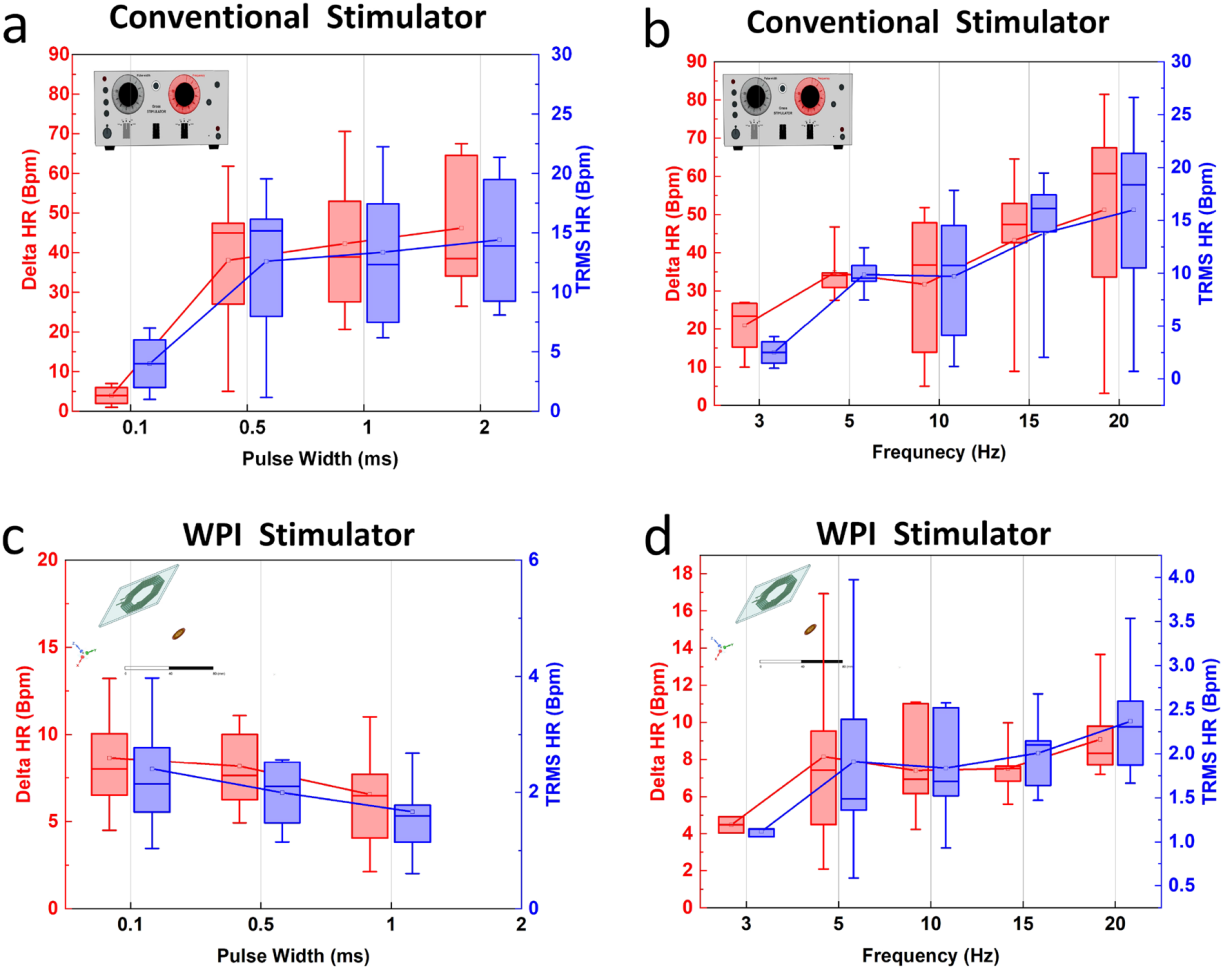
Comparison of conventional wired stimulators with WPI. (**a**) $HR_{Delta}$ and $HR_{TRMS}$ response to the conventional stimulator in frequency sweep. (**b**) $HR_{Delta}$ and $HR_{TRMS}$ response to the conventional stimulator in frequency sweep. (**c**) $HR_{Delta}$ and $HR_{TRMS}$ response to WPI in pulse width sweep. (**d**) $HR_{Delta}$ and $HR_{TRMS}$ response to WPI in frequnecy sweep.

When stimulating the vagus nerve using the wired conventional stimulator, stimulation frequency was significantly and positively correlated with $HR_{TRMS}$ (r = 0.473, p = 0.0095) and $HR_{Delta}$ (r = 0.505, p = 0.001). The pulse width to a lesser extent (compared to frequency) had a positive correlation with $HR_{TRMS}$ (r = 0.403, p = 0.029) and $HR_{Delta}$ (r = 0.363, p = 0.052). In the stimulation using the WPI stimulator, stimulation frequency was significantly and positively correlated with $HR_{TRMS}$ (r = 0.505, p = 0.0019) and $HR_{Delta}$ (r = 0.481, p = 0.003). However, there was no significant correlation between the pulse width and $HR_{TRMS}$ (r = $-$0.076, p = 0.66) or $HR_{Delta}$ (r = $-$0.066, p = 0.70) using the WPI stimulator. Due to the limited storage capacity and choice of filtering capacitor, a higher charge delivery efficiency for shorter pulses is expected. Since longer pulse widths require more charge to be transferred, higher storage and filtering capacitors are needed to avoid voltage drops during stimulation. The response of $HR_{Delta}$ and $HR_{TRMS}$ to frequency and pulse width sweeps using the conventional stimulator are presented in Fig. 6a, b, and their response to WPI is represented in Fig. 6c, d. The strong correlation of response with frequency can be observed in Fig. 6b, d. The average stimulation current in case of the WPI is 0.6 mA less than the conventional stimulator ,which is also one cause of weaker $HR_{Delta}$ and $HR_{TRMS}$ responses at the same frequency and pulse width. The pulse width response of WPI followed a different trend due to limited charge stored, and there was a weak correlation with the pulse width for both the conventional stimulator and WPI based on Fig. 6a, c. These results are consistent with Ref.^14^ which shows that high degree of bradycardia at fixed current can be evoked across four pulse widths when frequency is swept from 2 to 20 Hz.

## Discussion and conclusion

To enable long and robust wireless operation, a customized IC along with a double-layer flexible coil was utilized. The usage of discrete diodes and commercial micro-controllers can be the limiting factor for size and performance in some of the prior stimulator technologies^3,5,20^. The methodology to maximize the power transfer on the transmitter side and the novel tuning method for miniaturized implants can also be used as a basis for future wirelessly powered medical implants. The simulated SAR in this work was orders of magnitude lower than the safety limit which allows the range of the device to be easily extended by increasing the power.

The inductive near field coupling technique presented in this work addressed traditional challenges such as imprecise tuning and the need for large coil size^27,28,39,40^. In the case of far- and near-field coupling, the range of operation is comparable to the wavelength, and higher tissue attenuation at higher frequencies causes a shorter operation distance^3,20,21^. Ultrasound power transfer techniques, which rely on vibrations to transfer power, suffer from attenuation by obstructions such as bone and muscle and they require a gel to be applied on the skin and physical contact, which limits their use^24,41^. In Ref.^3^ authors showed VNS in a short distance of 1.5 cm while the transmitter is attached on the skin. In Ref.^24^ authors have shown ultrasound powering of the implants in rats. However, the need to operate at long distances within large animals, studying the frequency and pulse width sweeps and, comparison with conventional stimulators are not satisfied in prior works. The goals of our research are to provide more efficient and reliable power transfer to implants and to design code-activated chips to prevent false triggering in multi-site stimulation applications. This work has realized long and robust wireless operation and potential for chronic use should be studied in future. A portable and wearable battery powered Tx device would also be a major benefit for future long-term animal studies.

In summary, this article presents a new approach for designing WPT links for medical implants. The WPI powers at 13.56 MHz ISM band and has SAR 4 orders of magnitude below the safety limit. The range of operation while using 0.1 W of peak power is 50 mm and can be extended to 100 mm by using 1 W of power. The tissue impedance was verified before the stimulation, and hemodynamic responses in HR and LVP were observed. Wireless and batteryless VNS with frequency and pulse width sweeps are presented in this work. The significance and reproducibility of the results were verified and comparisons with conventional wired stimulators were drawn. The light weight cuff-type WPI can be easily used for stimulation of deep and compact tissue regions such as stimulation of sacral nerve or occipital nerve^3,42,43^. By simply choosing a different type of LED this work can be utilized for optical neuromodulation applications as well^20,44^. The proposed device can address key WPT challenges such as frequency tuning, volume, and misalignment, therefore, it opens up new possibilities for future wirelessly powered and controlled medical implants.

## Methods

### Wireless power link simulation

All the coupling, quality factor, misalignment, and SAR simulations were done using HFSS (Ansys Inc).

### Stimulator construction

The WPI is constructed using a customized microchip fabricated by TSMC standard 0.18 $\upmu$m CMOS technology and off-the-shelf surface mount components. The 12-turn coil is fabricated on a flexible 0.26 mm polyimide substrate. The coil has a simulated inductance of 2.93 $\upmu$H with a quality factor of 65.2. The chip is wire-bonded on the PCB, which is responsible for continuous power harvesting and demodulating the incoming data. Cuff electrodes and SMDs are used for the purpose of charge delivery to the tissue and charge balancing, respectively. The green LED on the PCB indicates the stimulation times. The construction of the implant requires the following components: (1) Customized chip, (2) Silver Epoxy, (3) Cuff electrodes(PerenniaFLEX Model 304, LivaNova PLC, London, United Kingdom), (4) Flexible polyimide PCB, (5) 22 $\upmu$F capacitor (AVX Corporation,04026D226MAT2A), (6) LED chip (Kingbright,APT1608LZGCK), (7) 47 k$\Omega$ resistor (Rohm Semiconductor,ESR01MZPJ473), (8) 10 $\upmu$F capacitor (AVX Corporation,04026D106MAT2A), (9) 47 pF capacitor (Murata Electronics,GCM1555C1H470FA16D).

In addition, the following tools are needed: (1) wire-bonding machine, (2) microscope, (3) hot plate, (4) silver epoxy (EPO-TEK,H20E), (5) tweezers, (6) blade, (7) biocompatible spectrally transparent epoxy (EPO-TEK, MED301), (8) needles.

PCB was constructed with a flexible polyimide substrate. After PCB construction, small amounts of silver epoxy were applied on pads using needles under a microscope as has been presented in Supplementary Fig. S2. In the next step, SMD components were put on the pads. The 22 $\upmu$F capacitor serves as energy storage. The LED indicates if the chip is stimulating or not. A 47 K$\Omega$ resistor discharges the residual charge on a 10 $\upmu$F filtering capacitor at the output. The PCB was then placed on a 180$^{\circ }$C hot plate for 30 min, which is presented in Supplementary Fig. S3, and afterward, epoxy was cured completely. The cuff electrode’s wire encapsulation was removed using tweezers and a blade. The wires were connected to two holes on the PCB using silver epoxy, and the PCB was reheated. Finally, the chip, SMD components, and exposed wires of cuff electrode were covered completely with biocompatible transparent epoxy, as presented in Supplementary Fig. S4.

### Impedance spectroscopy setup

For EIS measurement according to Fig. 4a, the PlamSense4 EIS device was connected to the two electrodes that were tightened around the nerves. The reference electrode (RE) and counter electrode (CE) were electrically shorted. The working electrode (WE) and RE were connected on two sides of stimulation electrode. The EIS scan equilibrium time is 4 s and impedance is measured from 1 Hz to 100 kHz using a 10 mV AC voltage.

### VNS setup

The RF signal generator (E4428C, Hewlett Packard Inc.) produces a 13.56 MHz signal, and pulse modulation of the same device defines pulse width and frequency of stimulation. The output of the RF signal generator can be connected to the power amplifier ( (ZHL-20 W-13 +, Mini-Circuits Inc.) for an extended operating range. The gain of the power amplifier is 50 dB.

### Surgical procedure and in vivo validation

*In vivo* studies were performed in three anesthetized male pigs (*Sus scrofa*) weighing 40-44 kg. Animal studies were approved by the University of California, Los Angeles Institutional Animal Care and Use Committee (IACUC). At the end of the study, animals were euthanized in accordance with the approved IACUC protocol and National Institutes of Health’s Guide for the Care and Use of Laboratory Animals. All methods reported are in accordance with Animal Research: Reporting of In Vivo Experiments (ARRIVE) guidelines.

Pigs were sedated with a mixture of tiletamine-zolazepam (5–8 mg/kg, intramuscular) and isoflurane (0.5–4%, inhaled). Animals were intubated and, mechanically ventilated, and vitals were monitored using 3-lead electrocardiography, pulse oximetry, arterial blood pressure, end-tidal carbon dioxide, and temperature. Arterial blood gas contents were evaluated hourly to ensure adequate physiologic status for experiments. Fentanyl (20 g/kg) was administered for analgesia during surgical preparation. A median sternotomy was performed to expose the heart. The right vagus nerve was exposed in the carotid sheath through an incision in the right lateral neck. A conductance catheter (Mikro-Tip, Millar Instruments, Houston, Texas) was placed in the left ventricle through the femoral artery and used to continuously measure heart rate and left ventricular pressure. Data were acquired using a data acquisition system (CED Model 1401, Cambridge Electronics Design, Cambridge, United Kingdom) and computed using Spike2 software (Cambridge Electronics Design, Cambridge, United Kingdom) for offline analysis.

Conventional wired stimulation was performed using bipolar vagus nerve stimulation electrodes (PerenniaFLEX Model 304, LivaNova PLC, London, United Kingdom). Electrodes were interfaced with a stimulator using a constant current photoelectric stimulus isolation unit (Grass S88 and PSIU6, Grass Instruments, Warwick, Rhode Island). Conventional stimulation was performed at varying frequencies (1–20 Hz), and pulse widths (0.1–1 ms). These parameters were selected to recruit parasympathetic efferent fibers and produce changes in cardiac parameters. Wireless stimulation was performed using the flexible implanted stimulator before and after incision closure. Device positioning was confirmed with fluoroscopy (GE OEC 9800 Plus C-Arm System). Wireless stimulation was similarly performed at constant voltage (3 V), varying frequency (1–20 Hz), and pulse width (0.1–1 ms).

### LVP data analysis

Data were imported into MATLAB (Math Works,MA), and heart rate was obtained from automated detection rising phase of LVP with a hysteresis of 0.1. For data analysis, heart rate during the prestimulus baseline periods ($-$10,000 to 0 ms) were segmented and compared to the stimulation period (0–10,000 ms). A 3rd order notch filter from 59 to 61 Hz was used to suppress the noise of the environment.

### EIS data analysis

The data from EIS were used to derive an equivalent circuit in PSTrace5.8 (PalmSense) circuit fitting. The circuit model was derived using the Levenberg-Marquardt algorithm with lambda start value of 0.01 and a scaling value of 10.

### Statistical analysis

Throughout the paper, data were presented as the mean and standard deviation (SD). A paired *t*-test was used to compare physiological responses to different vagus nerve stimulations. Comparisons were deemed to be statistically significant for *p* values $< 0.05$ for all analyses. The relationship between the HRV measurements ($HR_{TRMS}$, $HR_{Delta}$) and VNS settings (frequency, pulse width) was tested by Pearson’s correlation analysis, with $P < 0.05$ considered to be statistically significant. When box-and-whisker plots are displayed, the central line represents the median of the distribution, box limits represent the 25th and 75th quantiles, and whisker limits represent the full range of data. Preprocessing and data analysis were performed in MATLAB using custom-developed analysis scripts.

## Supplementary Information

Supplementary Information 1.

Supplementary Information 2.

Supplementary Information 3.

Supplementary Information 4.

## Data Availability

The datasets generated or reported in the reported studies and subsequent analysis are available from corresponding authors upon request.
