# Supplementary material for: Vagus nerve stimulation using a miniaturized wirelessly powered stimulator in pigs
Source: Sci Rep. 2022 May 17;12:8184. doi: 10.1038/s41598-022-11850-0 (PMC9114380; doi:10.1038/s41598-022-11850-0)
Supplement: Supplementary file 1 — Supplementary Information 1. [file 41598_2022_11850_MOESM1_ESM.docx]

**Supplementary Information:**

**Vagus Nerve stimulation using a miniaturized wirelessly powered stimulator in pigs**

Iman Habibagahi^1,*^, Mahmoud Omidbeigi^2,*^, Joseph Hadaya^3, 4, 5^, Hongming Lyu^1^, Jaeeun Jang^1^, Jeffrey L. Ardell^3, 4^, Ausaf A. Bari^2^, and Aydin Babakhani^1,*^

1. Electrical and Computer Engineering Department, University of California Los Angeles, Los Angeles, CA, USA
2. Department of Neurosurgery, University of California Los Angeles, Los Angeles, CA, USA
3. UCLA Cardiac Arrhythmia Center, University of California Los Angeles, Los Angeles, CA, USA
4. UCLA Neurocardiology Research Program of Excellence, University of California Los Angeles, Los Angeles, CA, USA
5. Molecular, Cellular and Integrative Physiology Program, University of California Los Angeles, Los Angeles, CA, USA

Corresponding Authors:

Iman Habibagahi: ihabibagahi@g.ucla.com

Mahmoud Omidbeigi: MOmidbeigi@mednet.ucla.edu

Aydin Babakhani: aydinbabakhani@ucla.edu

Fig. S1. Photos of the (a) backside and (b) front side of 13.56MHz transmitter coil.

Fig. S2. Photos illustrating (a) the usage of H20E 1Oz silver epoxy to assemble the SMDs on the PCBs and (b) applying silver epoxy via needle on golden pads and adjusting the SMD component on the pad.


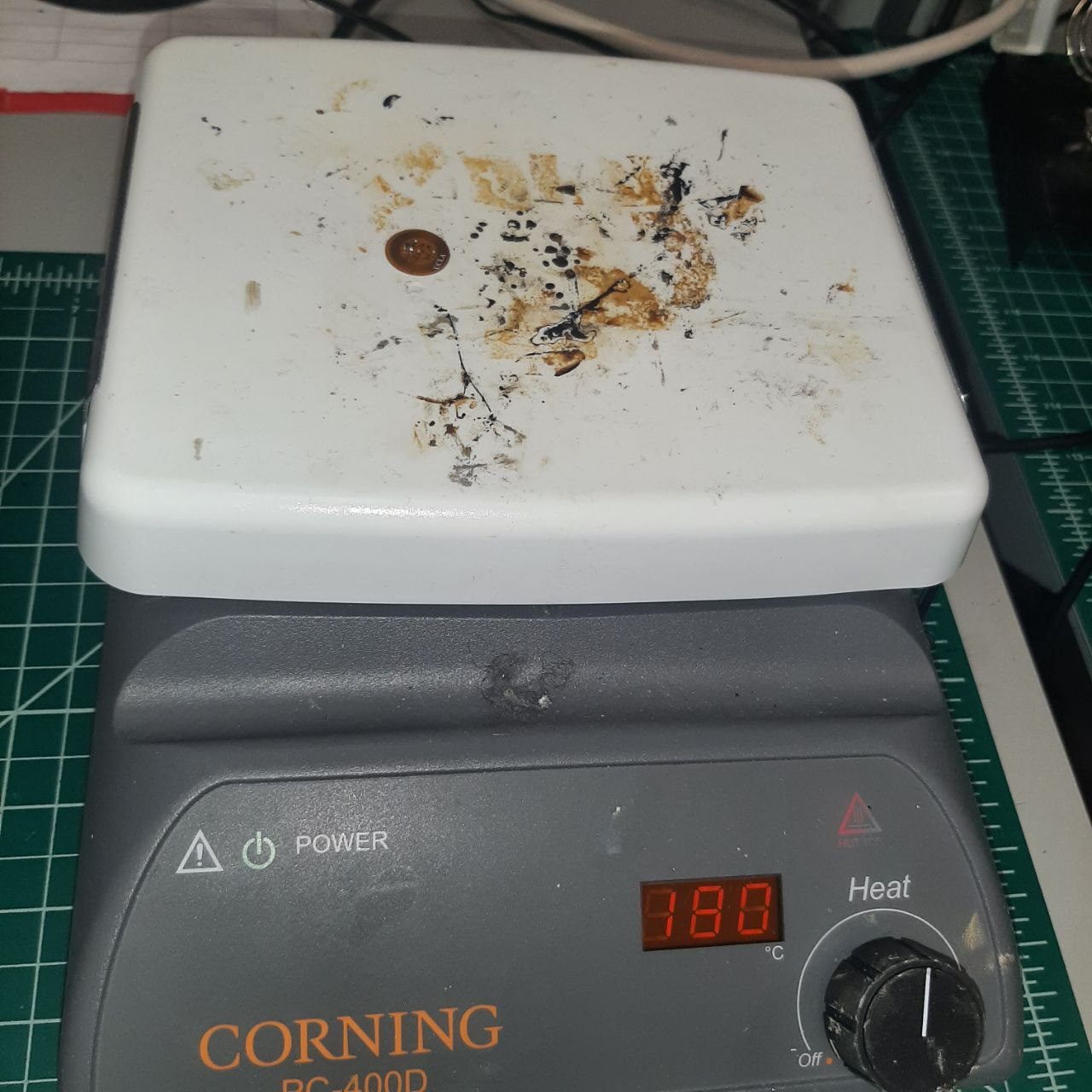


Fig. S3. Heating the PCB for 30mins at 180^◦^C in order to cure the epoxy and fixate the SMDs on the PCB.

Fig. S4. (a) Usage of MED-301-2FL biocompatible epoxy . (b) Applying biocompatible epoxy via foam swab on the surface of the PCB completely to make sure it has proper insulation from blood inside the animal.

**Video captions:**

Video S1. Setting up the carrier frequency (13.56MHz), power (0.1W), frequency (5Hz) and pulse width (0.1ms) on the external RF generator and response of the device before implanting to in 50 mm distance in air is observed.

Video S2. 5Hz stimulation using 0.1ms pulse width. The device is implanted in right side of the neck of the pig.

Video S3. Performance of the device in sutured wound, the change in heart rate (first line) is observed using Spike2 when the stimulation turns on.
